# ISN/RPS 2003 classification of lupus nephritis: time to take a look on the achievements and limitations of the schema

**DOI:** 10.12860/jnp.2014.17

**Published:** 2014-07-01

**Authors:** Muhammed Mubarak, Hamid Nasri

**Affiliations:** ^1^Department of Histopathology, Sindh Institute of Urology and Transplantation (SIUT), Karachi, Pakistan; ^2^Department of Nephrology, Division of Nephropathology, Isfahan University of Medical Sciences, Isfahan, Iran

**Keywords:** Lupus nephritis, Extracapillary proliferation, Antiphospholipid antibodies

## Abstract

*Implication for health policy/practice/research/medical education*:

Lupus nephritis (LN) is the most dreadful complication of systemic lupus erythematosus (SLE) and is responsible for the major share of morbidity and mortality of this disease. Its diagnosis, classification and management have posed significant challenges to the nephrologists and pathologists over the past several decades. A series of WHO classifications of LN were followed by the development of the international society of nephrology/renal pathology society (ISN/RPS) classification of LN in 2003. The classification has largely succeeded in achieving its goals, but a few limitations have also been exposed. It is time to revisit the classification in the light of experience of validation studies and new emerging data on this disease.

Lupus nephritis (LN) is the most feared and common complication of systemic lupus erythematosus (SLE) and is responsible for the major share of morbidity and mortality of this disease (1,2). Its diagnosis, classification and management have posed significant challenges to the nephrologists and pathologists over the past several decades (3-6). This editorial is focused on the strengths and weaknesses of international society of nephrology/renal pathology society (ISN/RPS) classification of LN (7). A series of WHO classifications of LN were followed by the development of the above classification in 2003 (8-10). The main objectives of this effort were to standardize the definitions, increase the reproducibility, remove the ambiguities of previous WHO classifications and to serve as the uniform language between the pathologists and between pathologists and nephrologists across the world (7,11). Since the publication of this classification, many studies have been carried out across different parts of the world to validate the classification in different settings and to test its reproducibility and the clinical relevance (12-21). Majority of these studies have found the classification useful in achieving its goals. However, a few shortcomings have also been exposed and it is time to revisit the classification in a systematic manner and revise it in the light of its weaknesses and new emerging data (6).

Now that almost 10 years have passed since the publication of this classification, it is right time to look back and ask; has the classification achieved the objectives set forth by its proponents? In other words, has the use of classification translated into improved patient outcomes, which is the ultimate goal of any classification scheme?

With respect to the above questions, the performance of the classification can be analyzed from several aspects; its comparison with previous WHO classifications, its prognostic value and clinical relevance, its shortcomings, and last but not the least, the need for its revision in response to the emerging molecular and omics data.

As regards the first objective of the classification, the superiority of the ISN/RPS classification over the previous WHO classifications is proved beyond doubt by a number of studies (12-14). The largest study comparing the two classifications was conducted by Furness and Taub and they showed that ISN/RPS classification has significantly higher interobserver concordance (12).

Regarding the prognostic and predictive value of the ISN/RPS classification, the studies have produced more conflicting results (15-21). This is particularly so concerning the studies focused on comparison between classes III and IV, specification of active and chronic lesions, and on subclassification of class IV. These are exactly the areas where interobserver concordance has been shown to be comparatively poor in studies (14).

Interestingly, although the validation studies have confirmed the laboratory and pathological differences between IV-G and IV-S subclasses, these studies have failed to confirm a significant difference in the outcome of these subclasses (15-21). Haring *et al*. in a meta-analysis of eight studies also did not find a statistically significant difference in the outcome between IV-G and IV-S subclasses (19). The reasons for this lack of correlation with outcome parameters are manifold. Different treatment regimens, follow-up intervals and different outcome parameters have been used in different studies. Even, the pathological definitions have differed among the studies (6).

The clinical relevance of the classification is also not proved beyond doubt. Although widely used in both clinical trials and clinical practice worldwide and endorsed by major collaborative groups, there is little evidence regarding its superiority over the previous WHO classification for this purpose (6).

The main shortcomings of the classification include its extremely “glomerulocentric” basis, lack of an evidence base, lack of specific incorporation of vascular and tubulointerstitial lesions and the lumping together in a dustbin manner of both active and chronic lesions in the same classes (6,22).

In the light of above deliberations, time has come for the proponents of the ISN/RPS 2003 classification to take note of the strengths and weaknesses of the classification and to revise the classification in the light of new data that has accumulated since the publication of this classification. Moreover, it is becoming increasingly important to rationalize the development of pathological classifications of different diseases (6). The development of the Oxford classification of IgA nephropathy (IgAN) represents a glaring example of the above approach and can serve as the role model for molding the other classifications to achieve the ultimate goals of a classification schema (23,24). Instead of creating artificial classes in this classification, specific pathological features that had independent prognostic value over and above the clinical and laboratory parameters at the time of biopsy or follow-up, are listed and their scoring given in a manner analogous to the listing of the real disease entities in the WHO classification of lymphoid malignancies (25). There is need for the formal inclusion of tubulointerstitial and vascular lesions and their scoring in the classification to further improve the prognostic value of the classification schema (26-30). The definitions of pathological lesions especially with regard to the activity and chronicity scoring also need further refinement to improve the reproducibility of these scores. It is perhaps right time to utilize the same strategy and approach for the classification of LN, which is also characterized by marked histological heterogeneity of the renal lesions on renal biopsy like IgAN.

In summary, the ISN/RPS 2003 classification of LN appears to have successfully achieved many of the objectives for which it was promulgated. Worldwide usage of the schema has exposed its strengths and limitations. So it is right time to revisit the classification in the light of the evidence accumulated from its validation studies and new emerging data on this disease.

## Authors’ contributions

MM and HN wrote the paper equally.

## Conflict of interests

The authors declared no competing interests.

## Funding/Support

None.
